# Output-Feedback Control for Discrete-Time Spreading Models in Complex Networks

**DOI:** 10.3390/e20030204

**Published:** 2018-03-19

**Authors:** Luis A. Alarcón Ramos, Roberto Bernal Jaquez, Alexander Schaum

**Affiliations:** 1 Posgrado en Ciencias Naturales e Ingeniería, Universidad Autónoma Metropolitana, Cuajimalpa, Mexico-City 05348, Mexico; 2 Departamento de Matemáticas Aplicadas y Sistemas, Universidad Autónoma Metropolitana, Cuajimalpa, Mexico-City 05348, Mexico; 3 Chair of automatic control, Kiel-University, Kiel 24143, Germany

**Keywords:** complex networks, discrete-time Markov-chain spreading models, feedback control

## Abstract

The problem of stabilizing the spreading process to a prescribed probability distribution over a complex network is considered, where the dynamics of the nodes in the network is given by discrete-time Markov-chain processes. Conditions for the positioning and identification of actuators and sensors are provided, and sufficient conditions for the exponential stability of the desired distribution are derived. Simulations results for a network of $N=10^{6}$ corroborate our theoretical findings.

## 1. Introduction

Modeling, analysis and control of the spreading of information and of infectious diseases have become a relevant problem of an interdisciplinary nature. Studies on spreading range from propagation of computer viruses in networks [1,2,3,4], epidemics in human populations [5,6,7,8,9,10,11,12], to spreading of rumors and information [13,14,15,16].

One of the early works for epidemic spreading [17] gave birth to the so-called SIRmodel that divides the population into three different compartments or groups: susceptible, infected and recover (or removed); and later, as many diseases do not confer any immunity, a simplified version of the SIR model was created, the so-called SIS (Susceptible-Infected-Susceptible) model, which has become one of the classical model of disease spreading. Although this model was proposed for modeling spreading in populations with no structure, nowadays, the SIS model has been extended to model the spreading process in a population mapped on a complex network.

In the field of computer virus propagation, Kephart and White [18,19] proposed one of the first models on networks, the so-called homogeneous model. This model is called homogeneous because every node of the network has the same probability of interaction with other nodes. Using a rate of infection and a death rate, they were able to calculate the infection threshold. Unfortunately, this model fails to capture real-world complexity. Data available show that real-world computer networks are not homogeneous and, instead, follow a power law structure in the number of connections of the nodes [20,21,22].

Another important method to study disease propagation is the so-called Degree Mean Field Approximation model (DMFA), which considers nodes with the same degree as dynamically equivalent [23]. Using this model, it is possible to calculate the epidemic threshold. Although this approach is quite interesting, it is not applicable in many realistic cases because nodes with the same degree do not necessarily behave the same way. Besides, the model does not provide information about the probability of individual nodes.

In recent years, Markov chain-based models for a Susceptible-Infected-Susceptible (SIS) dynamics over complex networks have been used [8,9,10,11,12,24]. Using these models, it is possible to determine the macroscopic properties of the system, as well as the description of the dynamics of individual nodes. One interesting result is that the calculated infection threshold depends on the value of the spectral radius of the adjacency matrix.

In spite of the fact that an increasing number of studies on the analysis of epidemic spreading have been published in the last decade, the studies dedicated to the control of these process are not very great in number. Different authors with interesting insights have been working on this subject, but at the end, we have three main categories for controlling complex networks (see also [25]): spectral control, optimal control and heuristic feedback control.

In the spectral optimization control methods, one of the goals is to make the eigenvalue λ(A) of the adjacency matrix *A* as small as possible. This could be achieved removing nodes from *A* that could be feasible by either immunizing or quarantining certain individuals [26]. The removal of links to reduce λ is also possible isolating cities or, more difficultly, restricting interactions among individuals.

In the optimization control methods for complex networks, the necessary and sufficient condition for spreading extinction poses an optimal control problem, where the curing rates are allowed to vary over time, depending on the evolving state of the system.

In [27], the linearization of the non-linear Markovian equation for the SIR model is studied around the extinction state and showed that the optimal solution is a bang-bang controller with at most one switch. This kind of control deserves further research, although, up to this date, it presents several flaws: it assumes that direct control over infection and recovery rates is always possible. These assumptions considered that these probabilities can be controlled for an entire population in an instantaneous way, which generally is unfeasible in a human population. This method is considered in different works [28,29].

In the heuristic feedback control extensions of the SIS and SIRS (Susceptible-Infectious-Recovered- Susceptible) model are considered. In this case, the model and its control are developed concurrently in such a way that the stability conditions can be derived. For example, in these models, we can have states corresponding to individuals that disconnect themselves from the net when they suspect that they have become infected (Protected state (P)). This means that control structures are already built in and available in the model itself in a heuristic fashion. As examples in the literature, we have the works of [30,31], which gives humans the possibility to take actions once they are in an Aware state (A). One of the flaws of these models is their great specificity.

In this work, we will study the spreading of information (diseases, rumors, gossip) using a Markov chain-based model for a Susceptible-Infected-Susceptible (SIS) dynamics over complex networks that has been used by different authors [8,9,10,11,12].

Using this model, we present a solution for the long-standing problem of epidemic spreading extinction. First of all, we present a model that generalizes the interaction among nodes and reproduces results after choosing some particular interaction. Afterwards, we determine sufficient conditions for stabilizing the spreading of information to the extinction state in complex networks of arbitrary topology. At the same time, these conditions give us a clue about how viruses are propagated, allowing us to identify the nodes that do not need any control to reach the extinction state and to distinguish them from the nodes that need to be controlled. Inspired by the ideas of control theory, we associate the set of nodes that do not need to be controlled (in order to reach the extinction state) with the zero dynamics of the system [32]. The set of nodes to be monitored and controlled are identified, as well, and a feedback control is applied to them in order to stabilize the extinction state. Accordingly, we have proven that the extinction state is an asymptotically-stable fixed point for the zero dynamics.

We performed numerical simulations using a scale-free complex network constructed as disused by Barabasi et al. [22] with one million nodes and show a complete agreement of the numerical results with our theoretical findings.

The remainder of this paper is organized as follows: Section 2 presents the problem statement and definitions. In Section 3, we determine sufficient conditions that allow us to identify the set of nodes that need to be controlled in order to reach the extinction state. In Section 4, we propose a linear feedback control to stabilize the system. Simulations and results are shown in Section 5. Finally, in Section 6, the conclusions are presented.

## 2. Problem Formulation

In this section, (i) the considered class of systems is introduced and the underlying model discussed, and (ii) the specific control problem addressed in this study is introduced.

### 2.1. System Class

Consider a network of *N* nodes described by an undirected graph G(V,E) with $V=\{v_{1},v_{2},\ldots ,v_{N}\}$ being the set of nodes and $E=\{e_{i,j}\}$ the connecting edges, together with the corresponding adjacency matrix $A=\{a_{ij}\}$ where $a_{ij}=1$ if (i,j)∈E, and zero otherwise. According to the graph G(V,E), the neighbors’ set $V_{i}$ of a node $v_{i}\in V$ is defined as:

(1) $$V_{i}=\left\{v_{j}\in V\;|\;a_{ij}=a_{ji}=1\right\}\subset V,$$

and the number of neighbors (or degree) is given by $N_{i}=\left|V_{i}\right|$.

**Figure 1:**
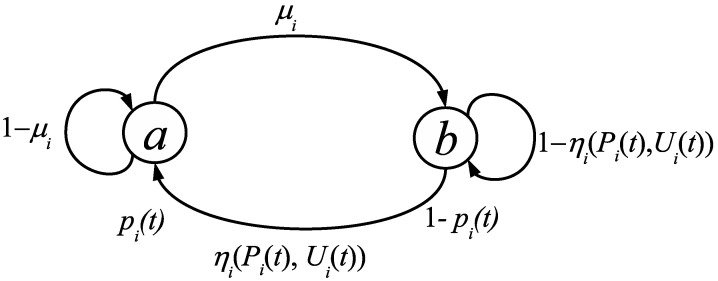
State transition diagram for a node $v_{i}\in V$.

The underlying process for every node is depicted in Figure 1, as a discrete time Markov process.

According to Figure 1, a node $v_{i}\in V$ can be in state *a* with probability $p_{i}\left(t\right)$ or in state *b* with probability $1-p_{i}\left(t\right)$, where $t\in N_{0}$ denotes the discrete time. The state *a* represents that the node $v_{i}$ has or knows the information to spread, and the state *b* represents the opposite. Any node $v_{j}\in V_{i}$ is able to spread information by interacting with some neighbor node $v_{i}$, but if the node $v_{j}$ does not know the information (i.e., $p_{j}=0$), then it does not contribute to spreading the information. Furthermore, each node has a manipulable variable $u_{i}\left(t\right)\in \left[0,1\right]$, which is amenable for feedback control.

The probability $p_{i}\left(t\right)$ is updated as follows: at each time step, every node $v_{i}$ can transit from *b* to *a* with probability $\eta_{i}\left(P_{i}\left(t\right),U_{i}\left(t\right)\right)$ under the influence of its neighbors, or transit from state *a* to *b* with a probability $\mu_{i}$, where:

$$0\leq \mu_{i},\eta_{i}\left(P_{i}\left(t\right),U_{i}\left(t\right)\right),p_{i}\left(t\right),u_{i}\left(t\right)\leq 1,\;i:v_{i}\in V,$$

and $U_{i}\left(t\right),P_{i}\left(t\right)$ are vectors such that:

$$\begin{array}{cc} U_{i}\left(t\right) & ={\left[\begin{array}{c} u_{i}\left(t\right),u_{k_{1}}\left(t\right),u_{k_{2}}\left(t\right),\ldots ,u_{k_{N_{i}}}\left(t\right) \end{array}\right]}^{T}\in P\left[N_{i}+1\right],\\ P_{i}\left(t\right) & ={\left[\begin{array}{c} p_{k_{1}}\left(t\right),p_{k_{2}}\left(t\right),\ldots ,p_{k_{N_{i}}}\left(t\right) \end{array}\right]}^{T}\in P\left[N_{i}\right],\;k_{l}:\;v_{k_{l}}\in V_{i}, \end{array}$$

with:

$$P\left[N\right]={\left[0,1\right]}^{N}.$$

The transition probability $\eta_{i}\left(P_{i}\left(t\right),U_{i}\left(t\right)\right)$ depends on the state of the neighbors given by $P_{i}\left(t\right)$ and a set of parameters $U_{i}\left(t\right)$ that are amenable to manipulation and models the interaction that spreads the information over $v_{i}$ due to its neighbor nodes. Note that despite the interaction between $v_{i}$ and its neighbor nodes, if $P_{i}=0$, then $\eta_{i}\left(P_{i},U_{i}\right)=0$ for any value of $U_{i}\in P\left[N_{i}+1\right]$, because in this case, the neighbor nodes do not know the information, and therefore, they do not contribute to spreading it.

Any change in $\eta_{i}\left(P_{i}\left(t\right),U_{i}\left(t\right)\right)$ with respect to $P_{i}\left(t\right)$ is assumed bounded and depends on $U_{i}\left(t\right)$ such that for each pair (i,j) where $i:v_{i}\in V$ and $j:v_{j}\in V_{i}$, there exists a function $m_{ij}\left(U_{i}\left(t\right)\right):P\left[N_{i}+1\right]\to R_{+}$ such that:

$$\left|\frac{\partial \eta_{i}\left(P_{i}\left(t\right),U_{i}\left(t\right)\right)}{\partial p_{j}}\right|\leq m_{ij}\left(U_{i}\left(t\right)\right),$$

or in terms of entries of the adjacency matrix:

(2) $$\left|\frac{\partial \eta_{i}\left(P_{i}\left(t\right),U_{i}\left(t\right)\right)}{\partial p_{j}}\right|\leq m_{ij}\left(U_{i}\left(t\right)\right)a_{ij}.$$

The above means that the transition probability η is a continuous function with bounded slope, which do not change abruptly.

Furthermore, throughout the paper, it is assumed that for all $P_{i}\in P\left[N_{i}\right]$, $\eta_{i}\left(P_{i},U_{i}\right)$ strictly monotonically increases with $u_{k}$, i.e.,

(3) $$\frac{\partial \eta_{i}\left(P_{i}\left(t\right),U_{i}\left(t\right)\right)}{\partial u_{k}}>0,\;\mathrm{for}\;k:\;v_{k}\in V_{i}\cup \left\{v_{i}\right\},$$

Finally, in order to be consistent with (3), it is assumed that:

(4) $$\frac{\partial m_{ij}\left(U_{i}\left(t\right)\right)}{\partial u_{k}}>0,\;\mathrm{for}\;i,j,k:\;v_{i}\in V,v_{j}\in V_{i},v_{k}\in V_{i}\cup \left\{v_{i}\right\}.$$

The dynamics for the probability $p_{i}\left(t\right)$ of node $v_{i}\in V$ to be in state *a* is given by (compare with [8,10,12]):

(5) $$p_{i}\left(t+1\right)=\left(1-\mu_{i}\right)p_{i}\left(t\right)+\eta_{i}\left(P_{i}\left(t\right),U_{i}\left(t\right)\right)\left(1-p_{i}\left(t\right)\right),\;p_{i}\left(0\right)=p_{i0},$$

or in vector form:

(6) $$P\left(t+1\right)=E_{\mu }P\left(t\right)+N\left(P\left(t\right),U\left(t\right)\right)\left(1-P\left(t\right)\right),\;P\left(0\right)=P_{0}$$

where P(t) is the system state, and U(t) the control parameters are given by:

$$\begin{array}{cc} P(t) & ={\left[p_{1}\left(t\right),p_{2}\left(t\right),\ldots ,p_{N}\left(t\right)\right]}^{T}\in P\left[N\right],\\ U(t) & ={\left[u_{1}\left(t\right),u_{2}\left(t\right),\ldots ,u_{N}\left(t\right)\right]}^{T}\in P\left[N\right] \end{array}$$

with 1 being a vector with unity entries and:

$$E_{\mu }=\mathrm{diag}\left(1-\mu_{i}\right),\;N\left(P,U\right)=\mathrm{diag}\left(\eta_{i}\left(P_{i},U_{i}\right)\right),$$

Additionally, for later use:

(7) $$\;M_{U}=\left\{m_{ij}\left(U_{i}\right)\right\},\;\mathrm{for}\;i:v_{i}:\in V,\;\mathrm{and}\;j:v_{j}\in V_{i}.$$

### 2.2. Control Problem

The purpose of the control design is to bring the state of the network to the extinction state. When the extinction state is not stable, the control must be designed in such a way that destabilizing components are compensated and self-stabilization mechanisms are enhanced. This can be accomplished in different ways, e.g., by changing the network structure [33,34,35] or node-specific parameters [27,36,37,38]. As for complex networks, not all node parameters are subject to control, and a systematic adaptation of parameters also implies computational complexity and may imply necessary communication structures, a set of nodes whose parameters should be adapted in order to achieve stabilization must be chosen. Once this set of nodes has been defined, it must be clarified what kind of adaptation mechanism provides the desired stabilization. A central question in this step is the decision about whether implementing a central computation structure, which decides on all parameters, or implementing a local, so-called decentralized control structure, which implies that the decision about how the control parameters are adapted, is computed locally at the node level. The implementation of this control on the other side requires some sensory information about the network state. Locating sensors in all nodes again implies high costs and complex communication structures, in particular if a centralized control structure is implemented that has to collect all the sensory data. Thus, a set of nodes for which sensor information must be provided in order to implement the control must be specified. In the sequel, the nodes for which sensor data are available are said to be monitored. A feedback control that depends on knowledge of the complete network state is called a state-feedback, while a control scheme that only requires the existing sensor information from the monitored nodes is called output-feedback control.

On the basis of the above discussion and terminology, the problem considered in this paper consists of designing a decentralized output-feedback control strategy to ensure the stabilization of the desired extinction state $P^{*}=0$ over the complex network G(V,E). In particular, this includes the determination of:

1. the set of *M* nodes that have to be monitored, i.e.,
  $$V_{m}=\left\{v_{m1},v_{m2},\ldots ,v_{mM}\right\},\;Y\left(t\right)={\left[\begin{array}{c} y_{1}\left(t\right),y_{2}\left(t\right),\ldots ,y_{M}\left(t\right) \end{array}\right]}^{T},\;y_{i}\left(t\right)=p_{mi}\left(t\right),\;v_{mi}\in V,$$
  where the number *M* has to be determined and Y(t) is the measurement vector,
2. the set of *K* nodes to be controlled, i.e.,
  $$V_{c}=\left\{v_{c1},v_{c2},\ldots ,v_{cK}\right\},\;v_{ci}\in V,$$
  where the number *K* has to be determined and,
3. the feedback control laws for the nodes $v_{i}\in V_{c}$
  (8) $$u_{i}\left(t\right)=\varphi_{i}\left(Y_{i}\left(t\right)\right)$$
  where $Y_{i}\left(t\right)\in R^{M_{i}}$ is the part of the measurement vector Y(t) that has to be accessible to node $v_{i}\in V_{c}$.

The approach for achieving this objective follows the constructive (passivity-based) control idea and consists of two steps: (i) assigning the necessary outputs so that the associated zero dynamics, i.e., the dynamics constrained to a submanifold:

(9) $$M_{0}=\left\{P\in P\left[N\right]\;|\;\forall \;v_{i}\in V_{c}:\;p_{i}=0\right\}$$

is asymptotically stable, and (ii) designing the controllers $u_{i}=\varphi_{i}\left(Y_{i}\right)$ so that for some 0≤γ<1, it holds that:

(10) $$p_{i}\left(t\right)\leq p_{i0}\gamma^{t}.$$

The decision about which nodes need to be measured will depend on the control laws $\varphi_{i}$ to be designed and is addressed in the subsequent analysis.

## 3. Selection of Monitored and Controlled Nodes

In this section, the central question about which nodes should be monitored in order to ensure a stabilization of the desired state probability distribution $P^{*}=0$ is addressed by determining a condition for exponential stability on the associated zero dynamics. Before analyzing this, notice that the fixed points associated with the dynamics (5) for some constant $U_{i}^{*}\in P\left[N_{i}+1\right]$ can be determined substituting the relation $p_{i}\left(t+1\right)=p_{i}\left(t\right)=p_{i}^{*}$. After some algebra, it follows that:

(11) $$p_{i}^{*}=\frac{\eta_{i}\left(P_{i}^{*},U_{i}^{*}\right)}{\mu_{i}+\eta_{i}\left(P_{i}^{*},U_{i}^{*}\right)}.$$

According to the above equation, we point out that the extinction state $P^{*}=0$ is a fixed point when $\eta_{i}\left(0,U_{i}^{*}\right)=0$; however, it is not clear if the extinction state or any other fixed point given by (11) is stable. The extinction state means that no information is spreading. In the virus spreading context, this condition plays an important role in order to explain how a virus is propagated.

The following lemma gives an important basis for the subsequent analysis.

**Lemma** **1.** *The set $P\left[N\right]={\left[0,1\right]}^{N}$ is positively invariant for the dynamics* (6)*.*

**Proof.**  Consider the case that for some $i:\;v_{i}\in V$, it holds that $p_{i}\left(t\right)=0$ for some t≥0. By (5), it follows that $p_{i}\left(t+1\right)=\eta \left(P\left(t\right),U\left(t\right)\right)\geq 0$. On the other hand, assume that $p_{i}\left(t\right)=1$ for some t≥0. It follows that $p_{i}\left(t+1\right)=\left(1-\mu_{i}\right)\leq 1$. Thus, for all $i:\;v_{i}\in V$, it holds that $0\leq p_{i}\left(t\right)\leq 1$ for all t≥0, showing that P[N] is a positively invariant set for the dynamics (6).  ☐

For the purpose of analyzing the stability properties, consider a constant $U^{*}={\left[u_{1}^{*},u_{2}^{*},\ldots ,u_{N}^{*}\right]}^{T}$ in the dynamics (6) with the fixed point given by (11). The following fundamental result on the stability of the origin of the dynamics (6) is obtained using a similar reasoning as in the analysis of epidemic spreading in [8].

**Lemma** **2.** *Consider the dynamics* (6) on a complex network with graph G(V,E) and adjacency matrix $A=\{a_{ij}\}$, and let $m_{ij}\left(U_{i}^{*}\right):P\left[N_{i}+1\right]\to R_{+}$ be a function satisfying (2) *and* (4)*. Then, the origin P=0 of the dynamical system* (6) is globally asymptotically stable if the constants $u_{i}^{*}$ are such that:

(12) $$\sigma \left(E_{\mu }+M_{U}\right)<1,\;U^{*}={\left[u_{1}^{*},u_{2}^{*},\ldots ,u_{N}^{*}\right]}^{T}\in P\left[N\right],$$

*where $E_{\mu }$ and $M_{U}$ are defined in* (7) *and σ(·) is the spectral radius of a matrix.*

**Proof.**  Using the mean value theorem [39] over $\eta_{i}\left(P_{i}\left(t\right),U_{i}^{*}\right)$ in order to proof the Lemma, one obtains:

$$\eta_{i}\left(P_{i}\left(t\right),U_{i}^{*}\right)-\eta_{i}\left(0,U_{i}^{*}\right)=\nabla_{P}\;\eta_{i}\left(\alpha P_{i}\left(t\right),U_{i}^{*}\right)\cdot P\left(t\right)\;\mathrm{for}\;\mathrm{some}\;\alpha \in \left[0,1\right],$$

where $\nabla_{P}$ denotes the gradient with respect to the vector *P*.

Recalling that when $P_{i}=0$, the neighbor nodes of $v_{i}$ do not contribute to spreading the information and, therefore, $\eta_{i}\left(0,U_{i}^{*}\right)=0$, it follows from the mean value theorem that:

(13) $$\begin{array}{cc} \eta_{i}\left(P_{i}\left(t\right),U_{i}^{*}\right) & =\eta_{i}\left(P_{i}\left(t\right),U_{i}^{*}\right)-\eta_{i}\left(0,U_{i}^{*}\right)=\nabla_{P}\;\eta_{i}\left(\alpha P_{i}\left(t\right),U_{i}^{*}\right)\cdot P_{i}\left(t\right)\\ & =\sum_{j\in V_{i}}\frac{\partial \eta_{i}\left(\alpha p_{i}\left(t\right),U_{i}^{*}\right)}{\partial p_{j}}p_{j}\left(t\right),\;\alpha \in \left[0,1\right]. \end{array}$$

Substituting the above equation into (5), one obtains:

(14) $$p_{i}\left(t+1\right)=\left(1-\mu_{i}\right)p_{i}\left(t\right)+\sum_{j\in V_{i}}\frac{\partial \eta_{i}\left(\alpha p_{i}\left(t\right),U_{i}^{*}\right)}{\partial p_{j}}p_{j}\left(t\right)\left(1-p_{i}\left(t\right)\right),\;\alpha \in \left[0,1\right].$$

Taking the absolute values on both sides of (14) yields:

$$\begin{array}{cc} |p_{i}\left(t+1\right)| & =\left|\left(1-\mu_{i}\right)p_{i}\left(t\right)+\sum_{j\in V_{i}}\frac{\partial \eta_{i}\left(\alpha p_{i}\left(t\right),U_{i}^{*}\right)}{\partial p_{j}}p_{j}\left(t\right)\left(1-p_{i}\left(t\right)\right)\right|\\ & \leq |1-\mu_{i}\left|\right|p_{i}\left(t\right)|+\sum_{j\in V_{i}}\left|\frac{\partial \eta_{i}\left(\alpha p_{i}\left(t\right),U_{i}^{*}\right)}{\partial p_{j}}\right||p_{j}\left(t\right)||1-p_{i}\left(t\right)|\\ & \leq \left(1-\mu_{i}\right)p_{i}\left(t\right)+\sum_{j\in V_{i}}\left|\frac{\partial \eta_{i}\left(\alpha p_{i}\left(t\right),U_{i}^{*}\right)}{\partial p_{j}}\right|p_{j}\left(t\right). \end{array}$$

By (2), the slope of $\eta_{i}$ is bounded, so it holds that:

$$p_{i}\left(t+1\right)\leq \left(1-\mu_{i}\right)p_{i}\left(t\right)+\sum_{j=1}^{N}m_{ij}\left(U_{i}^{*}\right)a_{ij}p_{j}\left(t\right).$$

Consider the auxiliary states $x_{i}\left(t\right)\geq 0,\;i=1,\ldots ,N$, so that $p_{i}\left(t\right)\leq x_{i}\left(t\right)$ and:

(15) $$x_{i}\left(t+1\right)=\left(1-\mu_{i}\right)x_{i}\left(t\right)+\sum_{j=1}^{N}m_{ij}\left(U_{i}^{*}\right)a_{ij}x_{j}\left(t\right).$$

These dynamics can be written in vector form as:

(16) $$X\left(t+1\right)=\left[E_{\mu }+M_{U}\right]X\left(t\right),$$

with $X\left(t\right)={\left[x_{1}\left(t\right),x_{2}\left(t\right),\cdots ,x_{N}\left(t\right)\right]}^{T}$ and $E_{\mu }$ and $M_{U}$ defined in (7). It follows that limX(t)=0 if and only if $\sigma (E_{\mu }+M_{U})<1$, i.e., all eigenvalues of $E_{\mu }+M_{U}$ are within the unit circle around the origin in the complex plane. As for all i=1,…,N, it holds that $p_{i}\left(t\right)\leq x_{i}\left(t\right)$, and it follows that $\lim_{t\to \infty }P\left(t\right)=0$ for all initial values $P_{0}\in P\left[N\right]$.  ☐

The asymptotic stability condition (12) is of a very general nature, given that it involves the complete network. One can refine it to gain insight into the conditions that every node $v_{i}\in V$ has to satisfy in order to ensure that the complete state P∈P[N] converges to $P^{*}=0$.

**Lemma** **3.** For a constant $U_{i}^{*}\in P\left[N_{i}+1\right]$ and for every $v_{i}\in V$, the state vector P(t)∈P[N] globally asymptotically converges to the desired state $P^{*}=0$ if:

(17) $$\forall \;v_{i}\in V:\;\sum_{j=1}^{N}m_{ij}\left(U_{i}^{*}\right)a_{ij}<\mu_{i}.$$

**Proof.**  The proposition is proven according to Gerschgorin’s theorem [40], which provides an upper-bound estimate for the spectral radius of a given matrix. For the matrix $E_{\mu }+M_{U}$, the theorem yields the following inequality:

$$\left|\lambda -(1-\mu_{i})\right|\leq \sum_{j=1}^{N}m_{ij}\left(U_{i}^{*}\right)a_{ij}$$

where λ represents an eigenvalue of matrix $E_{\mu }+M_{U}$. The above inequality is bounded as follows:

$$\begin{array}{c} \left|\lambda \right|-\left|1-\mu_{i}\right|\leq \left|\lambda -(1-\mu_{i})\right|\leq \sum_{j=1}^{N}m_{ij}\left(U_{i}^{*}\right)a_{ij}\\ \left|\lambda \right|\leq \left|\lambda -(1-\mu_{i})\right|+\left|1-\mu_{i}\right|\leq \sum_{j=1}^{N}m_{ij}\left(U_{i}^{*}\right)a_{ij}+\left|1-\mu_{i}\right| \end{array}$$

Now, the solution of (16) converges asymptotically to zero if |λ|<1. Therefore, we bound the above inequality as follows:

$$\left|\lambda \right|\leq \sum_{j=0}^{N}m_{ij}\left(U_{i}^{*}\right)a_{ij}+\left|1-\mu_{i}\right|<1$$

Given that for all $i:\;v_{i}\in V$, it holds that $\mu_{i}\leq 1$, a sufficient condition to ensure asymptotic convergence in (15), and therefore in (5), is given by:

$$\sum_{j=1}^{N}m_{ij}\left(U_{i}^{*}\right)a_{ij}<\mu_{i}.$$

This completes the proof.  ☐

Note that if this condition does not hold, it gives a hint about how to choose the nodes to be monitored. Under this hypothesis, it seems appropriate to consider the set of controlled nodes $v_{i}\in V_{c}$ as those nodes that do no satisfy the condition (17) and the monitored nodes as those neighbor nodes $v_{j}\in V_{i}$ of the controlled node $v_{i}\in V_{c}$ including the controlled node $v_{i}$. As we will show later, it is sufficient to take $V_{m}=V_{c}$.

Provided a controller exists, which steers the controlled nodes $v_{i}$ exponentially to $p_{i}^{*}=0$, the dynamics converges exactly to the submanifold $M_{0}$ defined in (9), called the zero dynamics [32,41]. By the control action, this manifold is a positively invariant subspace of P[N]. Furthermore, note that with the monitored nodes $p_{i}=0$, they no longer influence the spreading process, so that the zero dynamics correspond to a spreading process over a reduced network, from which the monitored nodes have been withdrawn. As the nodes included in this reduced network by construction satisfy Condition (17), the desired state vector $P^{*}=0$ is the unique attractor fixed point in $M_{0}$. This is summarized in the following corollaries.

**Corrollary** **1.** *If the nodes that do not satisfy the condition* (17) are controlled, then the zero-dynamics has $P^{*}=0$ as the unique asymptotically stable fixed-point.

Note that the establishment of the asymptotic stability of $P^{*}\in M_{0}$ for the zero dynamics is a key step in the constructive control approach [41], but does not yet ensure the asymptotic stability of $P^{*}\in P\left[N\right]$. This will be addressed later.

The condition (17), for a given node $v_{i}\in V$, establishes a relation between the amount of the interaction neighbors-to-node, with the properties of the node (given by $\mu_{i}$). A high interaction neighbors-to-node, regardless of the number of neighbors, can produce the spreading of information over the network. Thus, these nodes should be controlled.

On the other hand, it is possible to establish an alternative bounding dynamics for (5) in the same sense of Lemma 3, which yields the stability condition stated next.

**Lemma** **4.** For a constant $U_{i}^{*}\in P\left[N_{i}+1\right]$, the state vector P(t)∈P[N] globally asymptotically converges to the desired state $P^{*}=0$ if:

(18) $$\forall \;v_{i}\in V:\;\sum_{j=1}^{N}m_{ji}\left(U_{j}^{*}\right)a_{ji}<\mu_{i}.$$

**Proof.**  Consider the average probability of (5) and (13) as follows:

(19) $$\begin{array}{cc} \frac{1}{N}\sum_{i=1}^{N}p_{i}\left(t+1\right) & =\frac{1}{N}\sum_{i=1}^{N}\left[\left(1-\mu_{i}\right)p_{i}\left(t\right)+\eta_{i}\left(P\left(t\right),U_{i}^{*}\right)\left(1-p_{i}\left(t\right)\right)\right]\\ & =\frac{1}{N}\sum_{i=1}^{N}\left[\left(1-\mu_{i}\right)p_{i}\left(t\right)+\sum_{j\in V_{i}}\frac{\partial \eta_{i}\left(\alpha p_{i}\left(t\right),U_{i}^{*}\right)}{\partial p_{j}}p_{j}\left(t\right)\left(1-p_{i}\left(t\right)\right)\right],\;\alpha \in \left[0,1\right]. \end{array}$$

Taking the absolute value on the above equation and recalling (2) as in the proof of Lemma 2, it follows that:

$$\begin{array}{cc} \left|\frac{1}{N}\sum_{i=1}^{N}p_{i}\left(t+1\right)\right|=\frac{1}{N}\sum_{i=1}^{N}p_{i}\left(t+1\right) & =\frac{1}{N}\sum_{i=1}^{N}\left|\left(1-\mu_{i}\right)p_{i}\left(t\right)+\sum_{j\in V_{i}}\frac{\partial \eta_{i}\left(\alpha p_{i}\left(t\right),U_{i}^{*}\right)}{\partial p_{j}}p_{j}\left(t\right)\left(1-p_{i}\left(t\right)\right)\right|,\\ & \leq \sum_{i=1}^{N}\left[\left(1-\mu_{i}\right)p_{i}\left(t\right)+\sum_{j=1}^{N}m_{ij}\left(U_{i}^{*}\right)p_{j}\left(t\right)a_{ij}\right]. \end{array}$$

The last term in the above inequality includes the entries of the matrix $E_{\mu }+M_{U}$ and the state vector P(t) as follows:

$$\begin{array}{cc} \left[E+M_{U}\right]P\left(t\right) & =\left(\begin{array}{c} \left(1-\mu_{1}\right)p_{1}\left(t\right)+\sum_{j=1}^{N}m_{1j}\left(U_{1}^{*}\right)p_{j}\left(t\right)a_{1j}\\ \left(1-\mu_{2}\right)p_{2}\left(t\right)+\sum_{j=1}^{N}m_{2j}\left(U_{2}^{*}\right)p_{j}\left(t\right)a_{2j}\\ \cdots \\ \left(1-\mu_{N}\right)p_{N}\left(t\right)+\sum_{j=1}^{N}m_{Nj}\left(U_{N}^{*}\right)p_{j}\left(t\right)a_{Nj} \end{array}\right)\\ & =\left(\begin{array}{c} \left(1-\mu_{1}\right)p_{1}\left(t\right)+m_{12}\left(U_{1}^{*}\right)p_{2}\left(t\right)a_{12}+\ldots +m_{1N}\left(U_{1}^{*}\right)p_{N}\left(t\right)a_{1N}\\ m_{21}\left(U_{2}^{*}\right)p_{1}\left(t\right)a_{21}+\left(1-\mu_{2}\right)p_{2}\left(t\right)+\ldots +m_{2N}\left(U_{2}^{*}\right)p_{N}\left(t\right)a_{2N}\\ \cdots \\ m_{N1}\left(U_{N}^{*}\right)p_{1}\left(t\right)a_{N1}+m_{N2}\left(U_{N}^{*}\right)p_{2}\left(t\right)a_{N2}+\ldots +\left(1-\mu_{N}\right)p_{N}\left(t\right) \end{array}\right). \end{array}$$

Note that the summation in (19) is performed over each entry of the vector $\left[E_{\mu }+M_{U}\right]P\left(t\right)$, where *i* represents the *i*-th row entry. Therefore, it is possible to rearrange the above vector as follows:

$$\left[E_{\mu }+M_{U}\right]P\left(t\right)=\left(\begin{array}{c} \left(1-\mu_{1}\right)p_{1}\left(t\right)\\ m_{21}\left(U_{2}^{*}\right)p_{1}\left(t\right)a_{21}\\ \ldots \\ m_{N1}\left(U_{N}^{*}\right)p_{1}\left(t\right)a_{N1} \end{array}\right)+\left(\begin{array}{c} m_{12}\left(U_{1}^{*}\right)p_{2}\left(t\right)a_{12}\\ \left(1-\mu_{2}\right)p_{2}\left(t\right)\\ \ldots \\ m_{N2}\left(U_{N}^{*}\right)p_{2}\left(t\right)a_{N2} \end{array}\right)+\ldots +\left(\begin{array}{c} m_{1N}\left(U_{1}^{*}\right)p_{N}\left(t\right)a_{1N}\\ m_{2N}\left(U_{2}^{*}\right)p_{N}\left(t\right)a_{2N}\\ \ldots \\ \left(1-\mu_{N}\right)p_{N}\left(t\right) \end{array}\right)$$

Summing over each entry of the column vector, and later over each column, we have:

$$\begin{array}{cc} \sum_{i=1}^{N}\left[\left(1-\mu_{i}\right)p_{i}\left(t\right)+\sum_{j=1}^{N}m_{ij}\left(U_{i}^{*}\right)p_{j}\left(t\right)a_{ij}\right] & =\left(1-\mu_{1}\right)p_{1}\left(t\right)+\sum_{j=1}^{N}m_{j1}\left(U_{j}^{*}\right)p_{1}\left(t\right)a_{j1}\\ & +\left(1-\mu_{2}\right)p_{2}\left(t\right)+\sum_{j=1}^{N}m_{j2}\left(U_{j}^{*}\right)p_{2}\left(t\right)a_{j2}\\ & +\ldots \\ & +\left(1-\mu_{N}\right)p_{N}\left(t\right)+\sum_{j=1}^{N}m_{jN}\left(U_{j}^{*}\right)p_{N}\left(t\right)a_{jN}\\ & =\sum_{i=1}^{N}\left[\left(1-\mu_{i}\right)p_{i}\left(t\right)+\sum_{j=1}^{N}m_{ji}\left(U_{j}^{*}\right)p_{i}\left(t\right)a_{ji}\right]\\ & =\sum_{i=1}^{N}\left[\left(1-\mu_{i}\right)+\sum_{j=1}^{N}m_{ji}\left(U_{j}^{*}\right)a_{ji}\right]p_{i}\left(t\right). \end{array}$$

Summarizing:

$$\sum_{i=1}^{N}p_{i}\left(t+1\right)\leq \sum_{i=1}^{N}\left[\left(1-\mu_{i}\right)p_{i}\left(t\right)+\sum_{j=1}^{N}m_{ij}\left(U_{i}^{*}\right)p_{j}\left(t\right)a_{ij}\right]=\sum_{i=1}^{N}\left[\left(1-\mu_{i}\right)+\sum_{j=1}^{N}m_{ji}\left(U_{j}^{*}\right)a_{ji}\right]p_{i}\left(t\right).$$

Associating the corresponding terms for every node $v_{i}$, we obtain:

$$p_{i}\left(t+1\right)\leq \left[\left(1-\mu_{i}\right)+\sum_{j=1}^{N}m_{ji}\left(U_{j}^{*}\right)a_{ji}\right]p_{i}\left(t\right).$$

Consider the auxiliary states $w_{i}\left(t\right)\geq 0$, i=0,1,…,N, so that $p_{i}\left(t\right)\leq w_{i}\left(t\right)$ and:

(20) $$w_{i}\left(t+1\right)=\left[\left(1-\mu_{i}\right)+\sum_{j=1}^{N}m_{ji}\left(U_{j}^{*}\right)a_{ji}\right]w_{i}\left(t\right).$$

A condition to ensure asymptotic convergence to zero in (20) is given by:

$$\left|1-\mu_{i}+\sum_{j=1}^{N}m_{ji}\left(U_{j}^{*}\right)a_{ji}\right|<1.$$

Thus:

$$\sum_{j=1}^{N}m_{ji}\left(U_{j}^{*}\right)a_{ji}<\mu_{i}.$$

Hence, given that $p_{i}\left(t\right)\leq w_{i}\left(t\right)$, the above inequality is a sufficient condition to ensure asymptotic convergence in the dynamics (5).  ☐

The preceding result gives another criterion about how to choose nodes to be monitored. In this case, the set of controlled nodes $V_{c}$ contains those nodes $v_{i}$ that do not satisfy (18), but it is not clear which nodes have to be monitored. However, the vectors $U_{j}^{*}$ ($v_{j}\in V_{i}$) in (18) have something in common: all have the same entry $u_{i}^{*}$. Thus, in this case, it seems appropriate considering that $V_{m}=V_{c}$. Finally, the dynamics (20) establishes an alternative upper bound for (5).

In the same sense of the condition of Lemma 3, if the controlled nodes are chosen as those that do not satisfy the condition (18), then by construction, the zero-dynamics has the origin as the asymptotically-stable fixed point, and this is summarized in the following corollary.

**Corrollary** **2.** *If the nodes that do not satisfy the condition* (18) *are controlled, then the zero-dynamics is asymptotically stable.*

Note that the condition (18) has a similar interpretation as the condition (17). In this case, a high interaction node-to-neighbors, regardless of the number of neighbors, can produce information spreading over the network. Therefore, those nodes that do not satisfy the above condition should be controlled.

## 4. Feedback Control Design

In this section, the question is addressed about how to design the feedback control (8) for the nodes $v_{i}\in V_{c}$ so that $\lim_{t\to \infty }\left|p_{i}\left(t\right)-p_{i}^{*}\right|=0$. Up to this point, the dependency of the transition probability $\eta_{i}$ on the control input $U_{i}\left(t\right)$ has been neglected, given that $U_{i}\left(t\right)$ was considered as a set of constant parameters. For the nodes to be controlled, it is now supposed that the dependency of $\eta_{i}$ on $U_{i}\left(t\right)$ is of a certain structure, which allows one to explicitly determine a control law that steers the nodes $v_{i}\in V_{c}$ to their desired values $p_{i}^{*}=0$. Before addressing the control design step, the following helpful result is established.

**Lemma** **5.** Let $U_{i,1}={\left[u_{i,1},u_{k1,1},u_{k2,1},\ldots ,u_{kN_{i},1}\right]}^{T},U_{i,2}={\left[u_{i,2},u_{k1,2},u_{k2,2},\ldots ,u_{kN_{i},2}\right]}^{T}\in P\left[N_{i}+1\right]$ with $u_{k,1}\leq u_{k,2},\;k=i,1,\ldots ,N_{i}$ and $v_{i}\in V$, then $m_{ij}\left(U_{i,1}\right)\leq m_{ij}\left(U_{i,2}\right)$ for all j such that $v_{j}\in V_{i}$.

**Proof.**  By virtue of the mean value theorem, it holds that:

$$m_{ij}\left(U_{i,2}\right)-m_{ij}\left(U_{i,1}\right)=\sum_{k=1}^{N}\frac{\partial m_{ij}\left({\bar{U}}_{i}\right)}{\partial u_{k}}\left(u_{k,2}-u_{k,1}\right)a_{ik},$$

where ${\bar{U}}_{i}=U_{i,1}+\delta \left(U_{i,2}-U_{i,1}\right)$, δ∈(0,1). Recalling the condition (4), having $u_{k,2}\geq u_{k,1}$, it follows that:

$$m_{ij}\left(U_{i,2}\right)-m_{ij}\left(U_{i,1}\right)\geq 0.$$

This completes the proof.  ☐

On the basis of the above developments, the set $V_{c}$ of nodes to be controlled is determined either according to Corollary 1 or Corollary 2, i.e., those nodes that do not satisfy either (17) or (18). For both cases, a sufficient condition for the asymptotic stability of the closed-loop system is provided next.

**Theorem** **1.** *Consider the dynamics* (5) *where the set $V_{c}$ of nodes to be controlled is determined according either according to Corollary 1 or 2, i.e., $V_{c}$ is the set of those nodes that do not satisfy either* (17) *or* (18)*, respectively. Let $V_{m}=V_{c}$, i.e., all controlled nodes are monitored, and let for all $i:\;v_{i}\in V_{c}$ the value ${\bar{u}}_{i}$ be such that the condition* (17)* or *(18)*, respectively, holds true. If the controls $u_{i}\left(t\right)$ satisfy:*

$$0\leq u_{i}\left(t\right)<{\bar{u}}_{i},$$

*then $\lim_{t\to \infty }p_{i}\left(t\right)=0$.*

**Proof.**  Let ${\bar{U}}_{i}\in P\left[N_{i}+1\right]$ be such that for all nodes $v_{i}\in V_{c}$ whose control input appears in $U_{i}$, its value is replaced by ${\bar{u}}_{i}$. Given $u_{i}\left(t\right)\leq {\bar{u}}_{i}$, it follows from Lemma 5 that $m_{ij}\left(U_{i}\left(t\right)\right)\leq m_{ij}\left({\bar{U}}_{i}\right)$, and thus:

$$\sum_{j=1}^{N}m_{ij}\left(U_{i}\left(t\right)\right)a_{ij}\leq \sum_{j=1}^{N}m_{ij}\left({\bar{U}}_{i}\right)a_{ij}<\mu_{i},$$

$$\sum_{j=1}^{N}m_{ji}\left(U_{j}\left(t\right)\right)a_{ji}\leq \sum_{j=1}^{N}m_{ji}\left({\bar{U}}_{j}\right)a_{ji}<\mu_{i}.$$

The above inequalities satisfy Lemmas 3 and 4, respectively, so $\lim_{t\to \infty }p_{i}\left(t\right)=0$.  ☐

From Theorem 1, in order to stabilize the system (5) to the extinction state, it is necessary to design a feedback control $u_{i}\left(t\right)$, $v_{i}\in V_{c}$, that takes values below the upper-bound ${\bar{u}}_{i}$. This can be ensured, e.g., by the linear feedback control:

$$u_{i}\left(t\right)=\alpha {\bar{u}}_{i}\left(1-p_{i}\left(t\right)\right),\;\alpha \in \left(0,1\right).$$

## 5. Feedback Control Example

To corroborate our results, we design a feedback control in the model proposed by Gomez [11] in order to stabilize the system to the extinction state. This is a discrete-time Markov contact-based epidemic spreading model that establishes the probability of infection of each node. However, we do not consider reinfection in the same time step, and in contrast with Gomez’s model, each node has different values for the recovery probability (μ), infection rate (β) and contact probability (*r*).

The probability of infection of each node *i* at time t+1 is given by:

(21) $$\begin{array}{cc} p_{i}\left(t+1\right) & =\left(1-\mu_{i}\right)p_{i}\left(t\right)+\left(1-p_{i}\left(t\right)\right)\left(1-\zeta_{i}\left(t\right)\right),\\ \zeta_{i}\left(t\right) & =\prod_{j=1}^{N}\left(1-\beta_{i}r_{j}p_{j}\left(t\right)a_{ij}\right). \end{array}$$

where $\zeta_{i}\left(t\right)$ is the probability for node *i* to be not infected by any neighbor and $a_{ij}$ are the entries of the corresponding adjacency matrix. The network is described by the set of nodes *V*, and the set of neighbors of node *i* is given by $V_{i}$, $i:v_{i}\in V$. There are N=|V| nodes in the network, and each node *i* has a degree $N_{i}=\left|V_{i}\right|$. According to our framework, $\eta_{i}\left(P_{i}\left(t\right),U_{i}\left(t\right)\right)$ is given by:

(22) $$\eta_{i}\left(P_{i}\left(t\right),U_{i}\left(t\right)\right)=1-\zeta_{i}\left(t\right)=1-\prod_{j=1}^{N}\left(1-\beta_{i}r_{j}p_{j}\left(t\right)a_{ij}\right).$$

Suppose that the parameters that are amenable to manipulation are $\beta_{i}$ and/or $r_{i}$, i.e., we assume that it is possible to improve the health of the nodes or avoid a node performing several contact attempts with its neighbors. Accordingly, the entries of the vector $U_{i}\left(t\right)$ are given by the infection rate (β) and the contact probability (*r*), i.e.,

(23) $$U_{i}\left(t\right)=\left[\beta_{i},r_{k1},r_{k2},\ldots ,r_{kN_{i}}\right],\;\forall i\in V,\;\mathrm{and}\;kl:v_{kl}\in V_{i}.$$

The fixed points $p_{i}^{*}$ associated with the dynamics (21) are:

$$p_{i}^{*}=\frac{1-\prod_{j=1}^{N}\left(1-\beta_{i}^{*}r_{j}^{*}p_{j}^{*}a_{ij}\right)}{\mu_{i}+1-\prod_{j=1}^{N}\left(1-\beta_{i}^{*}r_{j}^{*}p_{j}^{*}a_{ij}\right)},$$

for some constant values $\beta_{i}^{*}$ and $r_{i}^{*}$. Note that $p_{i}^{*}=0$ is a fixed point that represents the extinction state, but its stability is unknown. However, Lemmas (3) and (4) give us sufficient conditions to ensure asymptotic stability in the extinction state of the system (21).

According to our definition for $\eta_{i}$, it is necessary to determine if $\eta_{i}$ satisfy conditions (2) and (4), i.e., we have to prove that $\eta_{i}$ strictly monotonically increases with $u_{k}$ and to find a $m_{ij}$ strictly monotonically increases with $u_{k}$.

Condition (2) can be established taking the derivative with respect to $p_{k}$ (k=1,2,…,N) as follows:

(24) $$\begin{array}{cc} \frac{\partial \eta_{i}\left(P_{i}\left(t\right),U_{i}\left(t\right)\right)}{\partial p_{k}} & =\frac{\partial }{\partial p_{k}}\left[1-\prod_{j=1}^{N}\left(1-\beta_{i}r_{j}p_{j}\left(t\right)a_{ij}\right)\right]\\ & =\beta_{i}r_{k}a_{ik}\prod_{j=1,j\neq k}^{N}\left(1-\beta_{i}r_{j}p_{j}\left(t\right)a_{ij}\right)\\ & \leq \beta_{i}r_{k}a_{ik}=m_{ik}\left(U_{i}\left(t\right)\right)a_{ik},\;\forall i\in V,\;k:v_{k}\in V_{i}. \end{array}$$

The above equation shows that $m_{ik}\left(U_{i}\left(t\right)\right)$ monotonically increases with $u_{k}$, and its arguments are reduced to $m_{ik}\left(U_{i}\left(t\right)\right)=m_{ik}\left(\beta_{i},r_{k}\right)$.

The second condition is proven taking the derivative with respect to $\beta_{k}$ (k=1,2,…,N):

$$\begin{array}{cc} \frac{\partial \eta_{i}\left(P_{i}\left(t\right),U_{i}\left(t\right)\right)}{\partial \beta_{k}} & =\frac{\partial }{\partial \beta_{k}}\left[1-\prod_{j=1}^{N}\left(1-\beta_{i}r_{j}p_{j}\left(t\right)a_{ij}\right)\right]\\ & =\left\{\begin{array}{cc} 0 & \mathrm{if}\;i\neq k\\ \sum_{l=1}^{N}r_{l}p_{l}\left(t\right)a_{il}\prod_{j=1,j\neq l}^{N}\left(1-\beta_{i}r_{j}p_{j}\left(t\right)a_{ij}\right) & \mathrm{if}\;i=k. \end{array}\right.\\ & \geq 0. \end{array}$$

and then with respect to $r_{k}$ (k=1,2,…,N):

$$\begin{array}{cc} \frac{\partial \eta_{i}\left(P_{i}\left(t\right),U_{i}\left(t\right)\right)}{\partial r_{k}} & =\frac{\partial }{\partial r_{k}}\left[1-\prod_{j=1}^{N}\left(1-\beta_{i}r_{j}p_{j}\left(t\right)a_{ij}\right)\right]\\ & =\left\{\begin{array}{cc} 0 & \mathrm{if}\;i\neq k\\ \sum_{l=1}^{N}\beta_{l}p_{l}\left(t\right)a_{il}\prod_{j=1,j\neq l}^{N}\left(1-\beta_{i}r_{j}p_{j}\left(t\right)a_{ij}\right) & \mathrm{if}\;i=k. \end{array}\right.\\ & \geq 0. \end{array}$$

In both cases, $\eta_{i}$ monotonically increases with β or *r*.

Using Lemmas (3) and (4), we can conclude that the system (21) globally asymptotically converges to the desired state $P^{*}=0$, if all nodes $v_{i}\in V$ satisfy any of the following conditions:

(25) $$\sum_{j=1}^{N}\beta_{i}r_{j}a_{ij}<\mu_{i},$$

or:

(26) $$\sum_{j=1}^{N}\beta_{j}r_{i}a_{ji}<\mu_{i}.$$

Note that the above conditions give us a hint about how to design a feedback control to stabilize the extinction state and to assign the output of the system:

(27) $$Y\left(t\right)={\left[y_{i}\left(t\right)\right]}^{T}={\left[p_{i}\left(t\right)\right]}^{T},\;\mathrm{where}\;v_{i}\;\mathrm{does}\;\mathrm{not}\;\mathrm{satisfy}\;(25),$$

or:

(28) $$Y\left(t\right)={\left[y_{i}\left(t\right)\right]}^{T}={\left[p_{i}\left(t\right)\right]}^{T},\;\mathrm{where}\;v_{i}\;\mathrm{does}\;\mathrm{not}\;\mathrm{satisfy}\;(26).$$

### 5.1. Simulations

We perform several simulations of the dynamical system (21) for different initial conditions, with the following considerations:

1. We incorporate preferential attachment in a network with $N=10^{6}$ nodes, according to [22]. To incorporate the growing character of the network, we started with a small number $m_{o}=9$ of vertices (linked randomly), and at every time step, we add a new vertex with m=3 edges until we reach $N=10^{6}$ nodes. In spite of the fact that we are using a scale-free network, we emphasize that our results are independent of the network’s topology.
2. The constant values for the recovery probability ($\mu_{i}^{*}$), infection rate ($\beta_{i}^{*}$) and contact probability ($r_{i}^{*}$) were distributed uniformly over the nodes with values into the interval [0.2,0.7].
3. In order to verify our results, we calculated the average probability as follows:
  (29) $$\rho \left(t\right)=\frac{1}{N}\sum_{i=1}^{N}p_{i}\left(t\right).$$

The system simulated is given by:

(30) $$\begin{array}{cc} p_{i}\left(t+1\right) & =\left(1-\mu_{i}\right)p_{i}\left(t\right)+\eta_{i}\left(P_{i}\left(t\right),U_{i}\left(t\right)\right)\left(1-p_{i}\left(t\right)\right),\\ \eta_{i}\left(P_{i}\left(t\right),U_{i}\left(t\right)\right) & =1-\prod_{j=1}^{N}\left(1-\beta_{i}r_{j}p_{j}\left(t\right)a_{ij}\right), \end{array}$$

where:

$$\left|V\right|=N=10^{6},\;N_{i}=\left|V_{i}\right|,\;\mu_{i}^{*},\beta_{i}^{*},r_{i}^{*}\in \left[0.2,0.7\right],$$

$$P_{i}\left(t\right)={\left[p_{k1}\left(t\right),p_{k2}\left(t\right),\ldots ,p_{kN_{i}}\left(t\right)\right]}^{T},\;U_{i}\left(t\right)={\left[\beta_{i}\left(t\right),r_{k1}\left(t\right),r_{k2}\left(t\right),\ldots ,r_{kN_{i}}\left(t\right)\right]}^{T},\;kl:v_{kl}\in V_{i},$$

$$0\leq p_{i}\left(t\right),\beta_{i}\left(t\right),r_{i}\left(t\right),\mu_{i}\left(t\right)\leq 1,\;\forall \;v_{i}\in V,$$

$$Y\left(t\right)={\left[p_{i}\left(t\right)\right]}^{T},\;v_{i}\in V_{ca}\;\mathrm{or}\;V_{cb},$$

where $V_{ca}$ is the set of those nodes *i* that do not satisfy (25) and $V_{cb}$ is the set of those nodes *i* that do not satisfy (26).

#### 5.1.1. Behavior of the System in the Absence of Control

**Figure 2:**
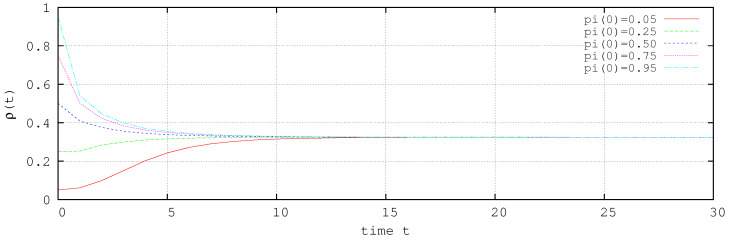
ρ(t) for several initial conditions without control.

Figure 2 shows the simulations results for the system (30) in the absence of control. The result shows that the system (30) presents an endemic state independent of the initial conditions, and it shows that about 30% of the nodes are probably infected.

Our results give us 495,091 nodes do not satisfy Condition (25) and 477,061 nodes that do no satisfy (26); therefore, we identify these nodes as the nodes to be controlled and monitored in order to steer the system to the extinction state.

#### 5.1.2. Behavior of the Nodes Associated with the Zero Dynamics

In order to corroborate that the associated zero dynamics is asymptotically stable, we set Y(t)=0, i.e., for those nodes that do not satisfy (25), we consider $\beta_{i}=0$, and for those nodes that do not satisfy (26), we consider $r_{i}=0$; the action of considering $\beta_{i}=0$ or $r_{i}=0$ is equivalent to unlinking the node *i*.

**Figure 3:**
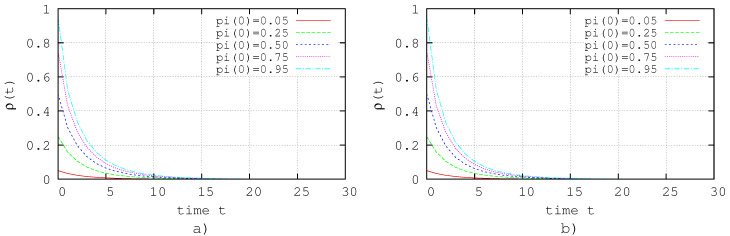
Simulation of the zero dynamics, i.e., without those nodes that do not satisfy (25) or (26). (**a**) Simulation of (30) with $\beta_{i}=0$ where $i:v_{i}\in V_{ca}$; (**b**) simulation of (30) with $r_{i}=0$ where $i:v_{i}\in V_{cb}$.

Under this conditions, the dynamical evolution of the system (30) is shown in Figure 3a,b. Note that, in both cases, the extinction state is reached after 15 time steps approximately.

#### 5.1.3. Behavior of the System with a Linear Feedback Control

We perform two separate simulations that will show the dynamical evolution of the system when a control is implemented. In the first one, we propose a linear control that will act only on those nodes that do not satisfy Condition (25). In the second one, a linear control will act only on those nodes that do not satisfy Condition (26). According to Theorem 1, we must establish an upper bound, in either case, namely ${\bar{\beta }}_{i}$ and ${\bar{r}}_{i}$, respectively. These upper bounds can be determined using Equation (25) or (26). For Condition (25), we have:

(31) $$\begin{array}{c} \sum_{j=1}^{N}{\bar{\beta }}_{i}r_{j}^{*}a_{ij}=\mu_{i}^{*},\\ {\bar{\beta }}_{i}=\frac{\mu_{i}^{*}}{\sum_{j=1}^{N}r_{j}^{*}a_{ij}}. \end{array}$$

and for Condition (26):

(32) $$\begin{array}{c} \sum_{j=1}^{N}\beta_{j}^{*}{\bar{r}}_{i}a_{ji}=\mu_{i}^{*},\\ {\bar{r}}_{i}=\frac{\mu_{i}^{*}}{\sum_{j=1}^{N}\beta_{j}^{*}a_{ji}}. \end{array}$$

Note that (31) relates the infection probability $\beta_{i}$ of the node *i* with its recovery probability $\mu_{i}$ and the contact capacity of its neighbor nodes, given by $\sum_{j=1}^{N}r_{j}^{*}a_{ij}$. On the other hand, Equation (32) relates the contact probability $r_{i}$ of the node *i*, with its recovery probability $\mu_{i}$ and the infection capacity of its neighbor nodes, given by $\sum_{j=1}^{N}\beta_{j}^{*}a_{ji}$. This means that a node with a high rate of contact can be infected with a great probability; besides, a node with weak neighbor nodes (those with a high probability of infection) can be a focus of infection, as is intuitively clear.

Thus, the controls proposed should be such that:

(33) $$\beta_{i}\left(t\right)<{\bar{\beta }}_{i}\;\mathrm{and}\;r_{i}\left(t\right)<{\bar{r}}_{i}.$$

It follows that for the nodes that do not satisfy (25), the control proposed could be given by:

(34) $$\beta_{i}\left(t\right)=\gamma {\bar{\beta }}_{i}\left(1-p_{i}\left(t\right)\right),$$

and for those nodes that do not satisfy (26):

(35) $$r_{i}\left(t\right)=\gamma {\bar{r}}_{i}\left(1-p_{i}\left(t\right)\right),\;0<\gamma <1.$$

Note that both controls above depend on the state of the node *i* given by $p_{i}\left(t\right)$ and the properties of its neighbor nodes given by ${\bar{\beta }}_{i}$ and ${\bar{r}}_{i}$. The value γ must be in the interval (0,1) in order to satisfy (33).

**Figure 4:**
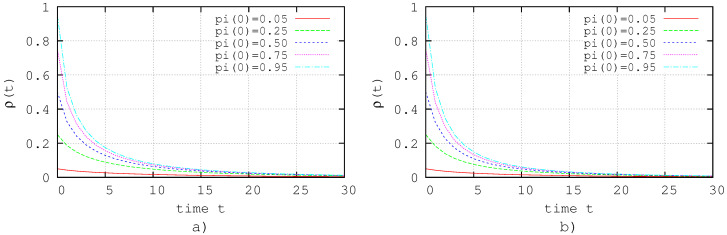
Linear feedback control. (**a**) Simulation with linear feedback control given by (34) where $i:v_{i}\in V_{ca}$; (**b**) simulation with linear feedback control given by (35) where $i:v_{i}\in V_{cb}$.

Figure 4a,b shows the results of the simulation of the system (30) with the applied controls (34) and (35), respectively, with γ=0.9. In both cases, it is shown that the extinction state is a closed-loop attractor, although not as fast as in the case of Figure 3a,b, corresponding to the evolution of the zero dynamics.

## 6. Conclusions and Outlook

A Markov chain-based model for a Susceptible-Infected-Susceptible (SIS) dynamics over complex networks has been analyzed, and a control mechanism has been proposed in order to stabilize the extinction state. Given that the system presents a high non-linear behavior, our analytical approach is based on determining a bilinear dynamics (15) and (20) that upper bounds the non-linear system. Following this approach, it is possible to determine two sufficient conditions (17) and (18) that ensure that the extinction state will be asymptotically stable. As a result of these conditions, we have determined which nodes are suitable for monitoring and controlling, in order to achieve the extinction of the propagation of the information. A linear feedback control scheme was tested for the stabilization of the extinction state in numerical simulation studies with $N=10^{6}$ nodes, showing the performance of the approach.

Future studies will consider generalizations of this approach to higher-dimensional node dynamics, multilayer networks [42,43], complex networks of agents [44,45] and analyzing the possibility of applying these kinds of control strategies for financial market models and decision dynamics [46,47,48].
